# QuoVidi: An open‐source web application for the organization of large‐scale biological treasure hunts

**DOI:** 10.1002/ece3.7130

**Published:** 2020-12-28

**Authors:** Guillaume Lobet, Charlotte Descamps, Lola Leveau, Alain Guillet, Jean‐François Rees

**Affiliations:** ^1^ Faculty of Bioengineering UCLouvain Louvain‐la‐Neuve Belgium; ^2^ Agrosphere Institute (IBG3) Forschungszentrum Jülich Juelich Germany; ^3^ Support en méthodologie et calcul statistique UCLouvain Louvain‐la‐Neuve Belgium; ^4^ Louvain Institute of Biomolecular Science and Technology School of Biology UCLouvain Louvain‐la‐Neuve Belgium

**Keywords:** active learning, biology, gamification, remote learning, systematics

## Abstract

Learning biology, and in particular systematics, requires learning a substantial amount of specific vocabulary, both for botanical and zoological studies. While crucial, the precise identification of structures serving as evolutionary traits and systematic criteria is not per se a highly motivating task for students. Teaching this in a traditional teaching setting is quite challenging especially with a large crowd of students to be kept engaged. This is even more difficult if, as during the COVID‐19 crisis, students are not allowed to access laboratories for hands‐on observation on fresh specimens and sometimes restricted to short‐range movements outside their home. Here, we present QuoVidi, a new open‐source web platform for the organization of large‐scale treasure hunts. The platform works as follows: students, organized in teams, receive a list of quests that contain morphologic, ecologic, or systematic terms. They have to first understand the meaning of the quests, then go and find them in the environment. Once they find the organism corresponding to a quest, they upload a geotagged picture of their finding and submit this on the platform. The correctness of each submission is evaluated by the staff. During the COVID‐19 lockdown, previously validated pictures were also submitted for evaluation to students that were locked in low‐biodiversity areas. From a research perspective, the system enables the creation of large image databases by the students, similar to citizen science projects. Beside the enhanced motivation of students to learn the vocabulary and perform observations on self‐found specimens, this system allows instructors to remotely follow and assess the work performed by large numbers of students. The interface is freely available, open‐source and customizable. Unlike existing naturalist platforms, allows the educators to fully customize the quests of interest. This enables the creation of multiple teaching scenarios, without being bound to a fixed scope. QuoVidi can be used in other disciplines with adapted quests and we expect it to be of interest in many classroom settings.

## Graphical abstract

QuoVidi is a decentralized web platform to organize large scale biological treasure hunts. It was created to teach students specific biological terms and observe them in their direct environments.

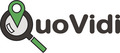

## 1 INTRODUCTION

Teaching biology to first‐year bachelor students is a challenge. As educators, our aim is usually threefold. First, we want the students to learn a new set of knowledge and integrate it. Second, and this is for us equally important, we want the students to engage with the topic at hand. We want to transmit our passion and curiosity about the topic that we teach. Third, we also want students to learn to observe the world around them. It is one thing to learn a topic from a textbook, it is another to observe it in real life. However, the main issue is that the classroom is, often by design, completely disconnected from the natural world. The challenge is therefore to find a way for students to learn and engage with biology, despite that given disconnection. Last but not least, in the Spring semester of 2020 (January–June) it was necessary for us to adapt the learning activities to the COVID‐19 crisis. Indeed, on 13 March 2020, the belgian government decided to impose a lockdown on the whole population, to contain the unprecedented pandemic. This situation was highly challenging from a teaching perspective. All the teaching activities, including practicals, designed to be given in person, at the university site, had to be modified on a short notice. The challenge was to maintain the teaching quality, despite the massive and sudden switch to remote learning.

The formal aim of our biology course—given in the Bioengineering Faculty, UCLouvain, Belgium—is to discover plant and animal structures, organs and their function at the individual scale. To achieve this, students need to learn specific vocabulary related to these structures. The classic way to present this vocabulary to a student audience is to review a series of slides illustrating these different characteristics. This vocabulary is usually very boring for teachers to describe (imagine the slides showing all the different shapes of leaves) and the content is not very interesting for students to listen to either. Yet this vocabulary is an important prerequisite for describing any biological structure and for later systematic identification of taxons using dichotomous keys. Its learning is essential. The question is therefore how to make this learning process motivating for the students and give them the opportunity to learn over time instead of memorizing a list of words? The additional difficulty is that this learning activity must be able to be set up with more than 300 students and few teaching resources.

To create this learning activity, we decided to draw inspiration from all the pedagogical techniques that aim to place the student at the center of their learning. Student‐centered learning and active learning emerged as important pedagogical techniques during the last century (Freeman et al., 2014). Active learning is characterized by (a) involving the student in the construction of his or her learning, (b) engaging the student in an in‐depth treatment of the subject matter, (c) constructing learning through interaction (with the teacher or other students), (d) conceiving of learning as the evolution of knowledge and skills (Chi et al., 2018; Freeman et al., 2014). Studies have shown that the more cognitively and socially engaged the student is in a learning task, the more perennial the learning task becomes (Chi & Wylie, 2014; Freeman et al., 2014). Active learning improves the performance of students and acts to reduce the achievement gap between advantaged and disadvantaged students (Haak et al., 2011). In order to stimulate learning through interaction and create a collective emulation around this activity, the idea of creating a campus‐wide biological treasure hunt finally emerged from the discussions. Beyond simply being active through the manipulation of information, the student has to transform and produce new information that is not provided in the learning material.

Gamification is another recent technique to better engage the students in a learning activity. Gamification is defined by (Kapp, 2012) as “game‐based mechanics, aesthetics, and game thinking to engage people, motivate action, promote learning, and solve problems.” In many studies, students' levels of engagement increased significantly following the introduction of game elements, such as points, challenges, quests, or progress bar (Alsawaier, 2018). The gamified environment can afford intrinsic motivation and engagement, which are also targeted by active learning.

To assemble these different elements—biological vocabulary, observation, active learning, and gamification—in a comprehensive learning activity, we created a large‐scale biological treasure hunt for our students. In short, we provided students with a list of specific biological vocabulary. They had to understand the list and find the different elements outside of the classroom, in the natural world. External resources (books, selected websites, wiki pages) describing this vocabulary were available to them. Complexity of understanding (some words are more difficult than others) as well as the difficulty of identification in the field were rewarded with different points.

To manage the treasure hunt, we designed a new web‐based platform, QuoVidi (which would loosely translate from latin as “where did you see”), for the organization of large scale, decentralized, biological treasure hunts. QuoVidi is an open‐source project available at www.quovidi.xyz. The objective of this publication is to describe the project, to show how we were able to adapt this learning activity to the COVID‐19 crisis, and finally, to show the results and success of the activity with the students.

## 2 PRESENTATION OF QUOVIDI

QuoVidi is a web application for the organization and management of large‐scale biological treasure hunts. It was created to teach students to learn new biological terms (both in zoology and botany) and to teach them to observe the natural world surrounding them. QuoVidi, unlike existing naturalist platforms, allows the educators to fully customize the quests of interest. This enables the creation of multiple teaching scenarios, without being bound to a fixed scope. Currently, the QuoVidi interface is available in English and French.

### 2.1 Setting up the activity

First, educators have to prepare a list of quests to find in the natural world. These quests should be tailored and adapted for the target public. For instance, in our experience with first‐year biology students, the quests revolved around biological structures and families (Table 1). Each quest is given a specific reward (points) depending on its intrinsic difficulty and rareness. Quests can be sorted in different groups (for instance “animal” and “plant”) and subgroups (for instance “animal species” and “leaf shapes”) to help students navigate them.

**TABLE 1:** Examples of quests used in the QuoVidi activity

Quest	Group	Subgroup	Points
Find an achene	Plant	Types of fruits	1
Find a flower with a bilateral symmetry	Plant	Types of flowers	2
Find a Siphonaptera	Animal	Animal groups	3
Find an example of aposematism	Animal	Animal physical attributes	1

Note

Quests are sorted in the different groups and subgroups to help students navigate them. Each quest yields a number of points depending on its difficulty.

Second, educators have to assign students to groups to perform the activity. Students in the same group will be able to share pictures and collaborate on the data collection. When logging into the web interface, students will be able to see the collected pictures and rewards from their own group. They will also be able to see the total number of points of the competing groups. Educators have access to the group data, as well as data from individual participants (e.g., number of submitted pictures). We decided to create a “group” layer in the interface, to increase the interactions between the students. Students in the same group will have to organize themselves and discuss the quest to fulfill the activity. It is worth mentioning that the groups also add a level of anonymity between the students, as only the name of the groups (that can be customized by the students) are visible in the charts.

Educators also have the possibility to define specific game parameters, such as specific geographic regions in which the game takes place or restriction on the number of submissions in each quest group (adding for instance a point penalty below a certain number of “animal” or “plant” submissions).

Once the list of quests, users, and groups are defined, the activity can start. Two main activities are available for the students: an in situ treasure hunt and an ex situ photograph quiz activity.

### 2.2 Treasure hunt

The main activity of the platform is the biological treasure hunt. Students have to go outside (although some of the creatures may be also found in their home such as food parasites, for example, *Lepisma* sp. or flies) to find the different quests setup by the educators. Once they find a specific quest, they have to take a picture of it with their smartphone. We ask the student to take unambiguous pictures, where the subject of the quest is clearly identified and visible. We also ask them to leave the natural environment intact, without killing any plant or animal in the process.

They can then store the picture on the QuoVidi web interface. When stored, pictures are automatically resized (for efficiency) and added to the activity database. Localization information and date are extracted from the picture EXIF metadata. Any other information is erased at this step, for privacy reasons.

Once pictures are stored on the web interface, students can assign them to a specific quest and submit it for evaluation. The web application allows users to follow their progress in detail (which picture was submitted for which quest, what is the evaluation status, etc.) as well as the global progress of the other groups (the total number of collected points).

It is worth noting that in Belgium—where the web application was first used—the lockdown due to the COVID‐19 pandemic still allowed citizens to go outside for short walks and exercises, although at a limited range. As such, the treasure hunt could still be performed by the students, either in their own garden or in neighboring areas. However, not everyone lives in the countryside or close to a natural environment, or had the opportunity to leave their home during the lockdown. This is why we created a second module in the interface, the photograph quiz, which allowed students to learn from photographs contributed by other students, without having to submit their own photographs.

### 2.3 Photograph quiz

The second module of the interface allows students to evaluate pictures submitted by other students (a modified version of peer evaluation). More precisely, in the photograph quiz module, students are presented with pictures submitted by other groups and validated by the educators (see below “Expert evaluation”). They have to assess whether the picture corresponds to its assigned quests. Their assessment is then compared to the assessment of the educators. If it matches, the students gain points that are added to their global group tally. An analysis of the performance of the student is presented in the “Results” section.

When performing this activity for the first time, it is necessary to have a sufficient amount of submitted (and corrected pictures). Without a database large enough, the activity loses some of its interest, as students might all review the same pictures.

### 2.4 Expert evaluation

The third important module of the interface, central to the activity, is the expert evaluation. Each submitted picture needs to be manually assessed by the educators. Different feedback can be given for each submission, such as “correct,” “correct and nice picture,” “incorrect,” “not visible” (e.g., the object is not visible in the picture) or “out of rules” (e.g., picture of a houseplant, picture taken outside of the prescribed geographical zones). The interface was designed to easily navigate the different quests and quickly correct the submitted images.

### 2.5 Comparison with existing tools

Over the past years, several online platforms have been created for the collection and identification of naturalist data. For instance, Pl@ntNet is focused on plant identification (Pl@ntNet Identify), eBird on birds (eBird), and iNaturalist on any living things (iNaturalist). These platforms often have features that have been designed for teaching (Teacher's guide iNaturalist).

The main difference between QuoVidi and these existing platforms reside in the flexibility of the scope. While the scope of iNaturalist or eBird is well defined (organism and species), educators can tailor the quests list in QuoVidi to fit their exact need. In our case, we wanted students to learn about biological structures, not yet about the species of plants and animals (which comes later in their training). QuoVidi allowed us to specifically choose the quests to fit our pedagogical goals.

### 2.6 Technical aspects of the web application

The web application was created using the R Shiny framework, using the shinydashboard (Chang & Borges, 2018), shiny (Chang et al., 2017), shinyWidgets (Perrier et al., 2019), shinyBS (Bailey, 2015), miniUI (Cheng, 2018) packages for the user interface design. The data are stored in a SQLite database, hosted on the server. The database management is done using the DBI (R Special Interest Group on Databases (R‐SIG‐DB) et al., 2018) and RSQLite (Müller et al., 2018) packages. Pictures are transformed and managed using the magick (Ooms, 2020) package. EXIF information is extracted using the exifr (Dunnington & Harvey, 2019) package. Data manipulation and visualization is done using the tidyverse (Wickham, 2017), lubridate (Grolemund & Wickham, 2011), cowplot (Wilke, 2019), formattable (Ren & Russell, 2016), DT (Xie et al., 2019), plyr (Wickham, 2009), leaflet (Cheng et al., 2018) packages. The text sentiment analysis was performed using the rfeel package (Abdaoui et al., 2017).

In our example, the web application was deployed on the university server with the following specifications: Ubuntu 18.04.4 LTS x86‐64, Linux kernel 4.15.0 x86‐64, R 3.6.2 x86‐64, Shiny server 1.5.12.933.

## 3 RESULTS

### 3.1 The web interface

The interface was created to be as user‐friendly as possible so that neither students nor staff need technical training. Because it is web based, it can be used on any platform, whatever the operating system. It scales on mobile devices as well, allowing users to store and submit pictures directly from the field (if they have an Internet connection).

**FIGURE 1:**
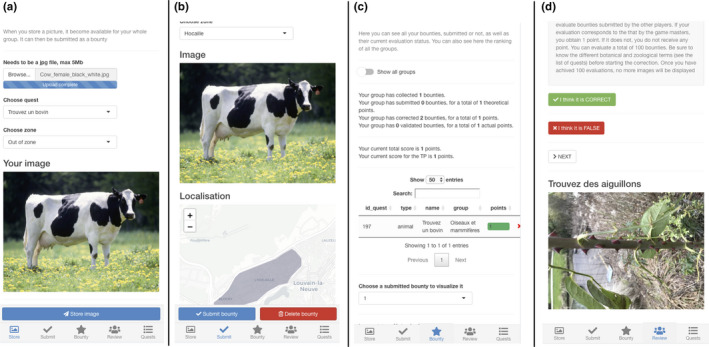
Overview of the different panels of the web interface. (a) Store panel, where students can load the pictures taken in the field into the interface. (b) Submit panel, where students can see all the stored pictures from their group and choose the ones to submit for evaluation. (c) Bounty panel, where students can track the progress of their group and the others, as well as the expert evaluation of their submitted images. (d) Review panel, where students can perform the photograph quiz module. Please note that the interface is available in French (as in the figure) and English

Figure 1 shows the different panels of the web interface. Figure 1a shows the “Store” panel, where students can store pictures, before submitting them for evaluation. This allows students from the same group to share and visualize their pictures. At this step, students can already assign a quest to the picture, which can be changed later on. They can also assign a geographic region, if this is required by the educators. A default region will be automatically proposed, based on the metadata of the picture.

Figure 1b shows the “Submit” panel. At this stage, students see all the pictures from their group. They can select a stored picture, assign it to a quest and submit it for evaluation. Groups can only submit one picture for each quest.

Figure 1c shows the “Bounty” panel, where students can visualize their progress. The panel presents an overview of the activity progress (for instance the total number of points or number of submitted quests). Students can also see the status of individual submissions, whether they are submitted or not as well as their validation status. In the same panel, students can also see the global scores of each group taking part in the activity. This adds a strong gamification aspect to the activity.

Figure 1d shows the “Quests” panel. In that panel, students can navigate through the different quests proposed by the educators. They can sort them by groups, subgroups, or rewards. In this panel, no explanation is given for the different quests. For instance, if the quest is “Find an achene,” we do not define achene. This is done by design. We want students to look up the different biological terms by themselves. We do provide them with resources to do so.

**FIGURE 2:**
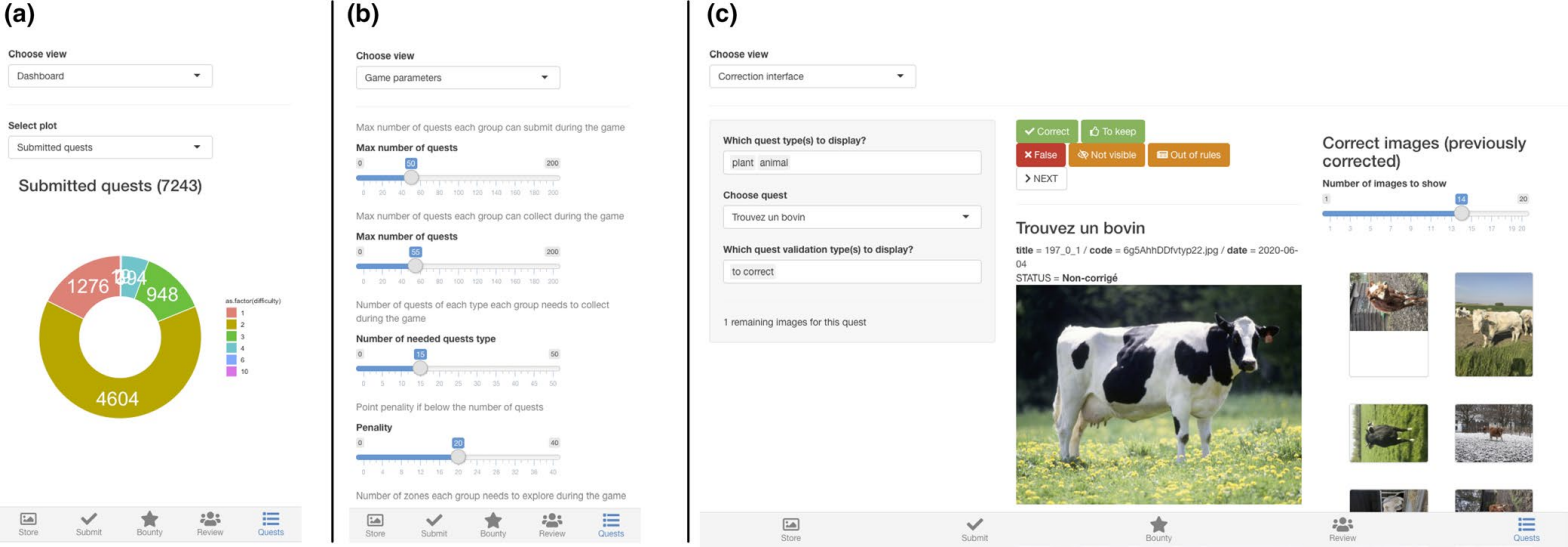
Overview of the different panels of the admin interface. (a) Overview of the advancement of the game. For instance, educators can see how many pictures have been submitted and which proportion of these pictures has been evaluated. (b) Game parameters. Educators can change the main game parameters directly through the web interface. (c) Correction interface. The correction interface was designed to allow a quick and efficient correction process by the educators. The educator chooses a specific quest to evaluate, then simply clicks on the relevant feed‐back button. The right panel shows examples of previously validated images

When an educator logs into the web application, the “Quests” panel becomes the “Admin” panel. In this panel, educators can follow the evolution of the activity (Figure 2a), change the activity parameters (Figure 2b) or correct the student submissions (Figure 2c). Depending on the number of participating students and allowed submissions, the number of corrections can quickly become quite large. Therefore we designed the corrections interface to be fast and efficient. The educator first chooses one quest to correct. He·She will be presented with submissions for that quest only. The corrections are done in one click, on the appropriate feedback button. Previously validated submissions for this quest are presented on the side panel, to help maintain the consistency of the evaluations. The validated pictures are also a useful help for educators with a lesser expertise. Our experience shows that it takes, on average, 5–10 s to evaluate one submission.

### 3.2 The 2020 activity

In Spring 2020, we organized the activity with a rooster of 346 first‐year bachelor students from the Bioengineering Faculty of the UCLouvain (Belgium). Students were spread in 346 groups (it was therefore set up as an individual activity). Although students had to do the activity individually, we encouraged them to discuss the different quests and collect them together, as long as everyone took their own pictures. Each group was allowed to submit a maximum of 50 pictures. 285 quests were created, divided in 175 plant quests and 110 animal quests.

Specific restrictions were added to the game. A minimal number of animal and plant quests had to be collected by each group. Groups were also asked to collect pictures in different zones and biotopes (Table 2) around the University campus, in Louvain‐la‐Neuve (Belgium).

**TABLE 2:** Description of the different zones defined for the 2020 activity

Zone name	Description	Area (km^2^)
Bois de Lauzelle	Woody area	4.85
Hocaille	Urban area	0.92
Lac	Lake area	0.18
Bruyères	Urban area	0.75
Bois des Rêves	Woody area	1.4
Lauzelle‐Centre	Urban area	0.8
Vieusart	Agricultural area	1.81
Biéreau‐Baraque	Urban area with communal gardens	1.0

Note

The total area of the game was 11.71 km^2^.

The activity started on February 11. We had to pause the activity for 20 days at the beginning of the lockdown due to the COVID‐19 crisis. During that pause, we implemented the peer evaluation in the web interface (it was not part of the interface initially). The activity resumed on the 3rd of April and finished on the 15th of May. For the second phase of the activity, during the lockdown, all restrictions (quests groups and zones) were lifted as many students had returned to their home far away from the campus.

At the end of the activity, we sent an anonymous feedback form to the students and received 125 answers.

### 3.3 Biological data collection

**FIGURE 3:**
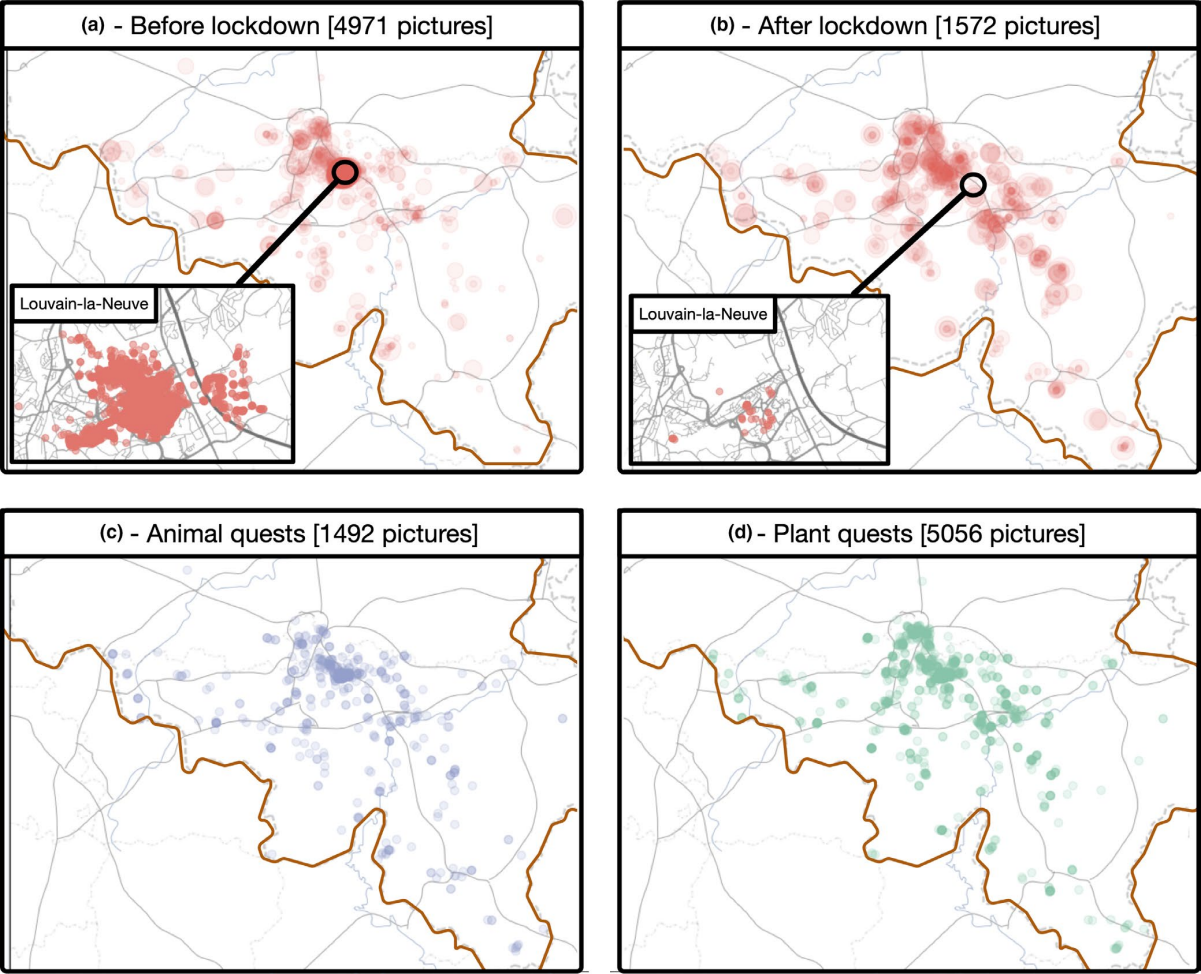
Overview of the data collected during the 2020 QuoVidi activity at the UCLouvain (Belgium). (a) Pictures collected before the lockdown due to the COVID‐19 crisis. (b) Pictures collected after the lockdown. (c) Animal quests collected during the whole activity. (d) Plant quests collected during the whole activity. The Belgian border is indicated in red

A total of 6,543 pictures were submitted by students during the 2020 activity. Figure 3 shows the repartition of the submitted pictures by the students during the activity. Figure 3a,b show the difference before and after the lockdown imposed during the COVID‐19 crisis.

Before the lockdown, as we asked students to take pictures around the university, most of them were taken in Louvain‐la‐Neuve. During the lockdown, almost no pictures were taken in Louvain‐la‐Neuve, as students went back home. The lockdown reduced the number of collected pictures, but did not stop it. This is due to several reasons. At the beginning of the activity, we encouraged students to look for quests in groups, to foster peer‐learning between them. This was not possible anymore during the lockdown. The collection of biological data was also influenced by the direct surroundings of the students. Students living in an urban area were potentially at a disadvantage compared to students in the countryside.

**FIGURE 4:**
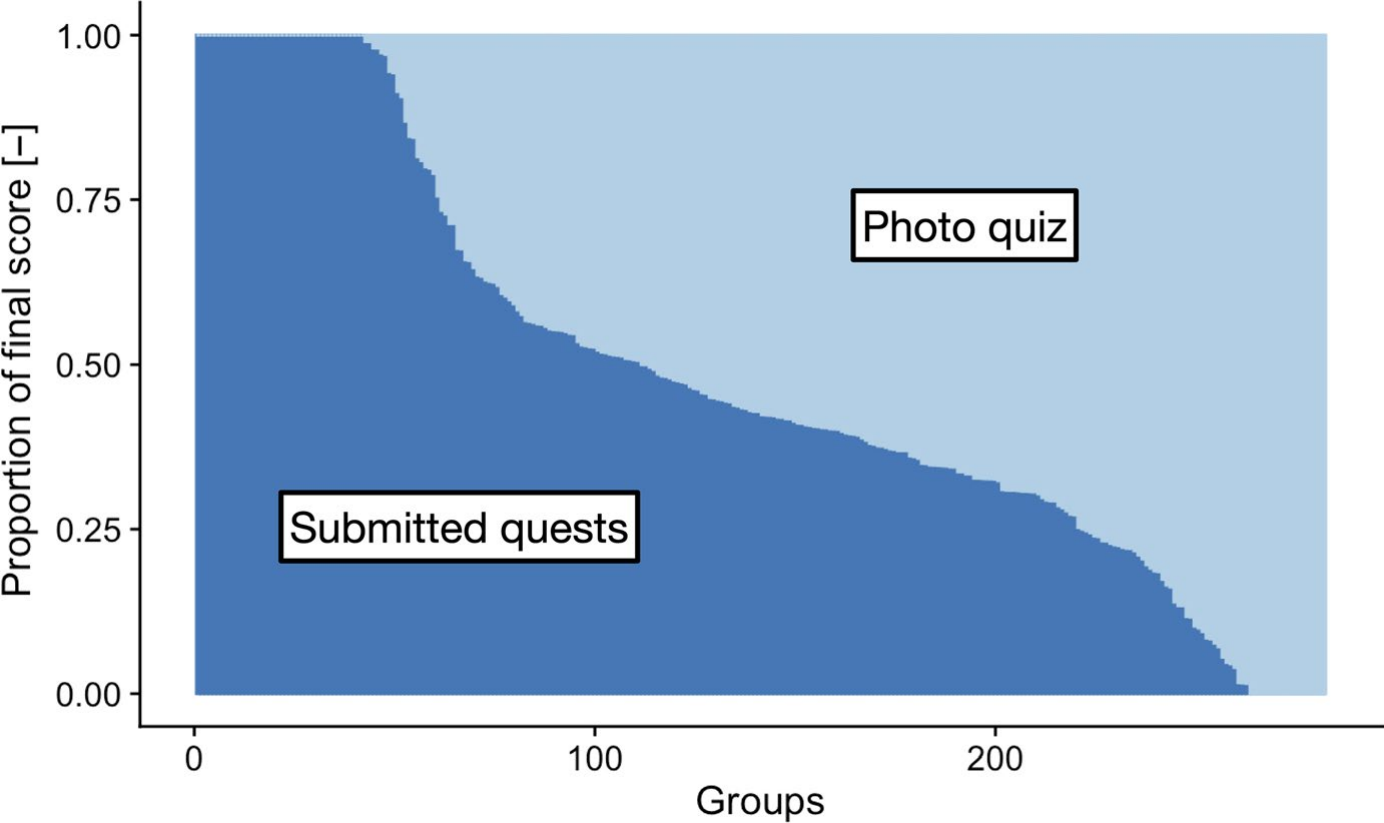
Proportion of submitted pictures and photograph quiz points for each group

However, because we included the photograph quiz module at the beginning of the lockdown, every student could continue the activity. Figure 4 shows, for every group, the proportion of points acquired either with the quests collection or the photograph quiz. We can see that the dual system allowed students to choose different strategies, to adapt to their individual lockdown conditions.

We also observed a strong trend toward the collection of plant‐related quests by the students (Figure 3c,d). This is probably due to the fact that, in an urban setting, plants are easier to find that animals. For an inexperienced naturalist, it is also probably easier to take pictures of plants than animals that have a tendency to escape. All the pictures can be viewed interactively at the address http://2020.quovidi.xyz

### 3.4 Student accuracy

**FIGURE 5:**
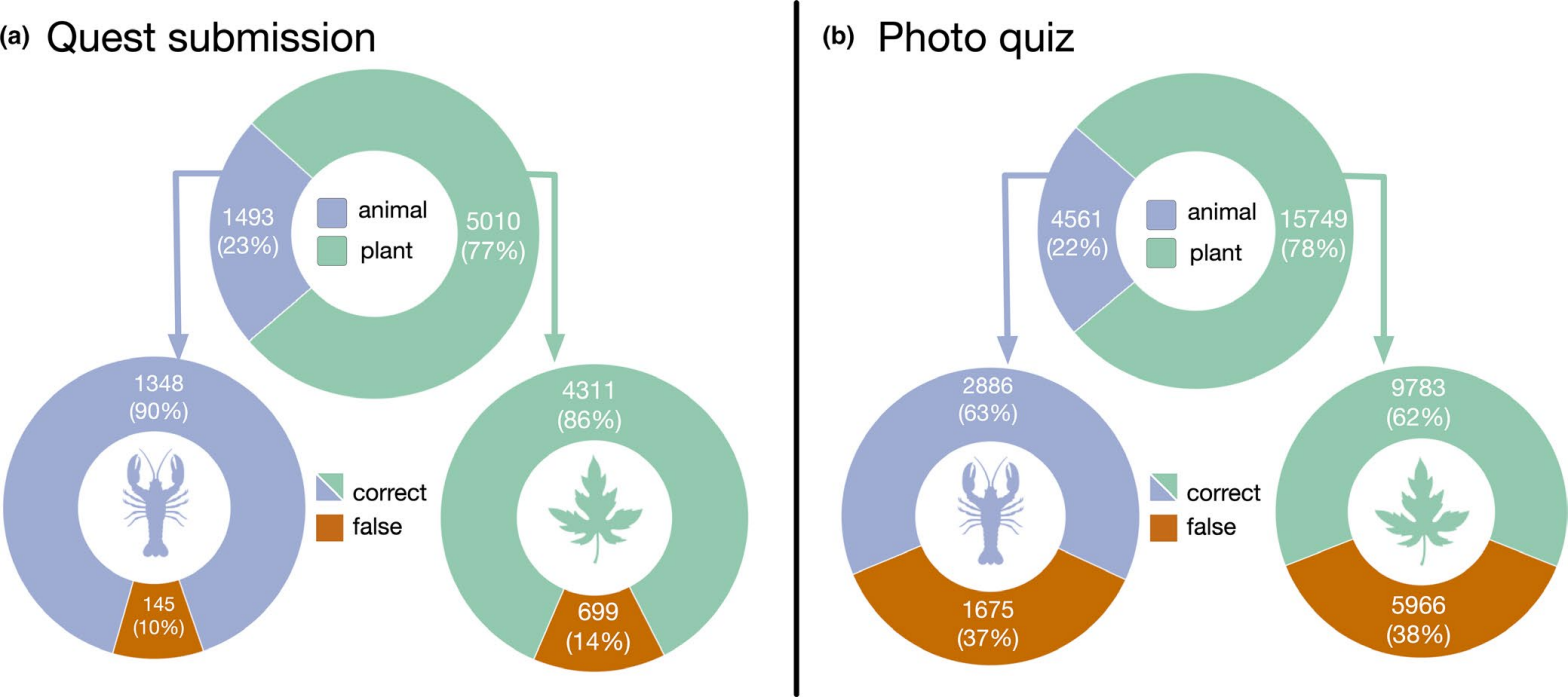
Performance of the students for the quest submission (a) and photograph quiz (b) activities. In each panel, the top chart represents the proportion of plant and animal quests. The bottom panels represent, for each type, the proportion of correct and false submissions/corrections

Overall, we observed a high correctness in the students picture submissions (Figure 5a). For the treasure hunt and the picture collection, only 10% and 14% of the quests (for the animal and plant, respectively) were assessed as incorrect by ourselves. One reason for such a high accuracy from the students might be the high level of engagement required by the activity. They have to learn the vocabulary and discuss with other students, and go outside often in groups to find what they have identified as appropriate for a quest submission. In the ICAP framework (Chi & Wylie, 2014), we believe this corresponds to the “Interactive learning” level, enabling the highest learning capabilities.

Interestingly, we also observed a much lower accuracy for the photograph quiz (Figure 5b). For that activity, 37% and 38% of the evaluations by the students (for the animal and plant, respectively) were incorrect. This can be due to several factors. First, contrary to the treasure hunt in itself, the evaluation activity requires a lesser level of engagement by the student. The activity is indeed “reduced” to click on a button in front of a computer screen. Second, depending on the quality of the picture to evaluate, said evaluation could be challenging. We tried to keep only good pictures for that activity, but the quality remained nonetheless variable.

### 3.5 Students feedback

At the end of the activity, we asked the students to fill an online feedback survey. One hundred and thirty‐seven students replied (40%). It should be noted that such direct feedback from the students should be taken with caution, as multiple factors, apart from the quality of the activity itself (educator gender, etc.) could influence the outcome of such evaluations (Stark & Freishtat, 2014).

**FIGURE 6:**
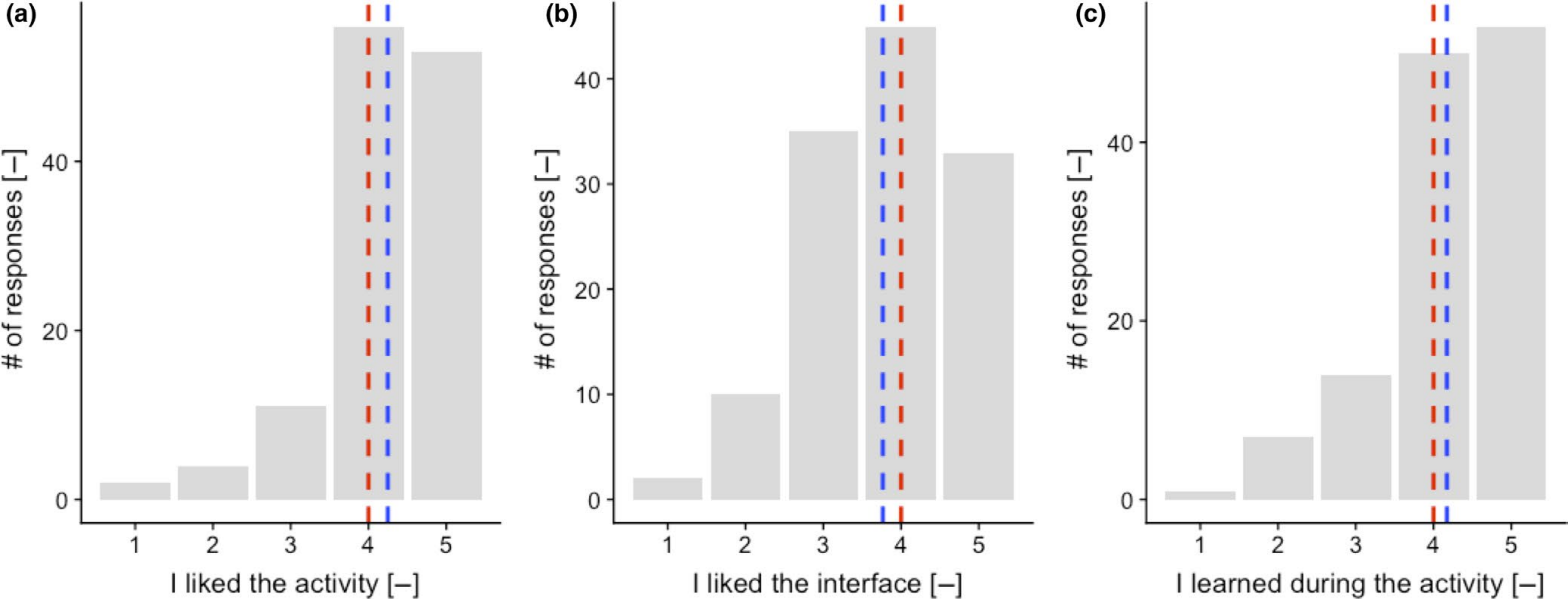
Feedback from the students. (a) Global appreciation of the activity by the students. (b) Appreciation of the web interface. (c) Self‐assessment of learning during the activity. The numbers on the *x*‐axis represent an increasing level of agreement with the statement presented, from strongly disagreed (1) to strongly agreed (5). Dashed red line represents the median while the dashed blue line represents the mean of the evaluation

Overall, the activity was very well appreciated by the students. With a few exceptions, students like going outside to observe their surroundings and collect the quests. 125 students (91% of the responses) reported to like the activity and have the feeling to have learned during it (Figure 6). Many students spontaneously expressed their enthusiasm for this activity (Table 3). The few negative comments revolved around three themes: The activity takes too much time; students do not always have the feeling to learn doing the activity; and the interface could be improved.

**TABLE 3:** Selected comments from the students received with the feedback form

**Selected positive comments from the students**
“Great activity to learn new concepts and look at our environment in a different way.”
“I think the game is fun and interactive, it's a great way to learn by seeing things "in real life" and also to decipher the quests.”
“Very nice way to propose the course, it pushes the students to discover the surrounding nature in a playful way.”
**Selected negative comments from the students**
“Improving the interface would make the activity more enjoyable”
“I think we will quickly forget what we have learned”
“The activity takes a lot of time”

## 4 DISCUSSIONS AND PERSPECTIVES

### 4.1 Remote learning through a centralized game

The QuoVidi platform was created for several reasons. We wanted students to learn and know specific plant and animal vocabulary, but we did not want to just give them a list of words to be memorized and repeated. We also wanted them to explore and learn to observe their direct environment. We wanted to show them that you do not need to go to a tropical forest to be able to see a great diversity of plant and animal forms and species. We wanted to spark a strong interest in their surrounding natural world. Finally, we were also working with strong practical constraints. We needed to design an activity that was scalable for hundreds of students, without the need to increase the number of educators. This was possible, thanks to the current technologies (camera, mobile network, and GPS localization) available in almost every mobile phone.

With the creation of the web platform for QuoVidi, we have met all those goals. The treasure hunt (and to a lesser extent the photograph quiz) strongly motivates students to learn and remember the different technical terms used in the quests. Then they have to apply these new terms directly in the field. The gamification process (quests, score points, personal progress panel, and scoreboard between all the groups) is also a strong incentive for the students to engage in the activity (Alsawaier, 2018). We believe this is especially the case for an activity that is usually not well viewed by the students, such as learning a large amount of technical vocabulary. We are planning to better quantify the relevance and importance of the gamification aspect in QuoVidi in the future.

The activity is also highly scalable. The number of participants is, from a technical point of view, only limited by the capacity of the server on which the platform is installed. The main limitation remains the expert correction step. As every single picture needs to be validated, the evaluation can quickly require a lot of time from the educator, even though we tried to make the process as efficient as possible. We hope in the future that the platform would benefit from advances in artificial intelligence algorithms to help correct the images (see below).

Finally, the activity is completely decentralized, which has been a great asset during the COVID‐19 crisis. Students can collect quests at any time and place, making it easy to adapt to every individual situation. If they cannot go outside, or are not in a nature‐rich environment, they can still participate in the activity via the peer evaluation module. From the educator point of view, all the management and corrections can be done from anywhere, as long as they have access to a computer and an Internet connection. As such, the platform was a real asset during the lockdown period (13 March to 8 of June in Belgium), as it enabled us to continue the activity almost seamlessly.

### 4.2 Improving the activity and the platform

Although students have largely appreciated the activity, we did receive constructive negative comments from several of them. Such comments will be useful to better use the platform and improve it in the future.

One comment that was often made by students, is that the activity takes a lot of time. Students might spend a whole afternoon looking for quests without finding many. Over‐enthusiastic students might also be tempted to spend too much time collecting quests. We believe this could be improved by better tailoring the size of the activity (how many quests we ask each group to collect), as well as with better communication with the students (explain more explicitly what is expected and not expected from them).

A second recurring comment by the students is the feeling that they do not truly learn (or at least not on the long term) by doing this activity. This is a comment often made by students in activity‐learning settings, although the final achievement is better than with passive learning (Deslauriers et al., 2019; Freeman et al., 2014). Again, we believe a better communication with the students about the pedagogical goal of the activity is a way to reassure them. Another strategy could be to couple the activity with a final evaluation.

Finally, students have made comments regarding the improvement of the platform itself. Without going into the details, such comments are continuously taken into account to improve QuoVidi. We also have the feeling that first‐year bachelor students do not always have the habit to use custom‐made websites. In the age of smartphones and easy to use mobile applications, the comparison could be difficult.

### 4.3 Reusing the image database

Similarly to citizen science projects, the use of our platform allows the collection of large numbers of geotagged, dated images of plant and animal structures. By helping create such a database over the years, the students are taking an active role in creating a valuable research and teaching resource that could be reused elsewhere. This in itself is viewed by the students as a motivational element of the activity. However, educators should be mindful of the local regulations and rules relative to data privacy and reuse. By default, the data collected during the game is not made public.

Such databases could be reused in different ways. From an educational point of view, the images collected could be used to create a quiz to rehearse the vocabulary the following year. The student would therefore create their own teaching and rehearsal material. An example of a quiz created with the students pictures is visible here: http://quiz.quovidi.xyz. We also plan to use the database created this year to feed the photograph quiz module of our next activity.

From a research point of view, an ever‐growing database of annotated plant and animal pictures (describing either organ, species, or groups), on a limited and well‐defined area would be a valuable resource. As each record of the database has been validated by an expert (the educators), such a database could be used in research projects.

Another interesting valuation of the database would be to reuse it to train deep learning recognition algorithms. Again, given the size and potential growth of the database, it will be an interesting resource to train machine learning models to recognize plant and animal structures. Such models could, in turn, be integrated into the platform to help with the correction.

### 4.4 Tackling plant awareness disparity

Plant awareness disparity is defined as the “inability to see or notice the plants in one's environment and therefore the inability to recognize the importance of plants in the biosphere and in human affairs” (Parsley, 2020; Wandersee & Schussler, 1999). In the general population, as well as within a student group, it translates in a lesser interest in plant studies, compared to animal studies (Wandersee & Schussler, 1999). This phenomenon is thought to have several societal impact, ranging from plant conservation and biodiversity (Balding & Williams, 2016) to food production (Knapp, 2019; Kritzinger, 2018).

Involuntarily, our teaching activity with QuoVidi helped tackle, to some extent, this phenomenon. Indeed, although the list of quests was balanced between animals and plants (60% of plant‐related quests), we observed a strong bias toward plant submissions by the students (77% of plant‐related submissions). As discussed earlier, this is probably due to several factors. First, plants are easier to spot, when you look for them, than animals. They do not move or run away. In addition, even in an urban setup, plants can be seen almost everywhere. A second factor relates to the cooperation between groups. Once a group has located a plant structure, it is relatively easy to communicate its position to other groups, as it is likely to stay in the same location. This is obviously not the case with animals.

### 4.5 Collaborations between groups

So far, we use the QuoVidi framework within a single classroom (even if it was a very large one). Since the activity is entirely centralized online, we could imagine collaboration between remote classrooms. Students from different regions, countries, or continents could participate in the same activity, hence, increasing the degree of diversity of the observations.

### 4.6 Expanding to new disciplines

Here, we exemplified the use of our platform with a biological treasure hunt. Students were asked to find, in the field, plant, and animal structures. However, due to its flexibility, the platform could be used to organize large‐scale treasure hunts in any context.

It could be used in architecture, design, or geology classrooms, with quests related to different building structures, street art, or rock, respectively. It could be used with children, with simplified quests, or with more advanced students, with more complex ones. In short, we expect the concept could be used in any context to deal with structures present in the “outside” world.

## 5 CONCLUSIONS

We presented in this manuscript a new open‐source web platform for the organization for large treasure hunt, QuoVidi.

During the Spring 2020, in the midst of the COVID‐19 crisis, we successfully used the QuoVidi platform with more than 300 students and allowed the collection of more than 6,000 geotagged plant and animal pictures. The decentralized nature of the platform enabled us to ensure a continuity in our teaching, despite the nation‐wide lockdown.

We expect QuoVidi to be of interest for any teaching activity focused on the identification of real‐world structures. QuoVidi is available at the address http://www.quovidi.xyz

## CONFLICT OF INTEREST

The authors declare that they do not have any conflict of interest.

## AUTHOR CONTRIBUTION

**Guillaume Lobet:** Conceptualization (equal); Data curation (equal); Formal analysis (lead); Methodology (equal); Resources (equal); Software (lead); Supervision (equal); Visualization (lead); Writing‐original draft (lead); Writing‐review & editing (equal). **Charlotte Descamps:** Conceptualization (equal); Methodology (equal); Writing‐original draft (equal); Writing‐review & editing (equal). **Lola Leveau:** Conceptualization (equal); Methodology (equal); Writing‐original draft (equal); Writing‐review & editing (equal). **Alain Guillet:** Software (supporting); Writing‐original draft (supporting). **Jean‐François Rees:** Conceptualization (equal); Methodology (equal); Resources (equal); Supervision (equal); Writing‐original draft (equal); Writing‐review & editing (equal).

### OPEN RESEARCH BADGES

This article has been awarded Open Data and Open Materials Badges. All materials and data are publicly accessible via the Open Science Framework at http://www.doi.org/10.5281/zenodo.3909033 and https://doi.org/10.5281/zenodo.4293690.

## Data Availability

QuoVidi is an open‐source project, released under an APACHE licence (Apache License, 2004). Everyone is free to reuse and modify it, with attribution. Project website: http://www.quovidi.xyz. Source code: https://doi.org/10.5281/zenodo.4293690. Script and data used for the manuscript: http://www.doi.org/10.5281/zenodo.3909033
